# Supplementary material for: Dynamic Magnetoelectric Effect of Soft Layered Composites with a Magnetic Elastomer
Source: Polymers (Basel). 2023 May 10;15(10):2262. doi: 10.3390/polym15102262 (PMC10220663; doi:10.3390/polym15102262)
Supplement: Supplementary file 1 [file polymers-15-02262-s001.zip › polymers-2375030-supplementary.pdf]

Table S1. The values of the fitting lines slope with errors and their R-square coefficients for the magnetic field dependences of the induced voltage

IP concentration	56 wt%	65 wt%	70 wt%	75 wt%	80 wt%
Slope	0.048±0.011	0.153±0.018	0.193±0.018	0.231±0.034	0.243±0.023
R-Square	0.78	0.93	0.96	0.90	0.95

Table S2. The values of the fitting lines slope with errors and their R-square coefficients for the magnetic field dependences of the resonant frequency

IP concentration	65 wt%	70 wt%	75 wt%	80 wt%
Slope	-0.003±0.001	-0.0110±0.0005	-0.014±0.003	-0.013±0.002
R-Square	0.45	0.98	0.87	0.92
